# Research and Applications of Additive Manufacturing in Oil and Gas Extraction and Gathering Engineering

**DOI:** 10.3390/ma18143353

**Published:** 2025-07-17

**Authors:** Xiang Jin, Jubao Liu, Wei Fan, Mingyuan Sun, Zhongmin Xiao, Zongheng Fan, Ming Yang, Liming Yao

**Affiliations:** 1 School of Mechanical and Aerospace Engineering, Nanyang Technological University, 50 Nanyang Avenue, Singapore 639798, Singapore; 13653458637@163.com (X.J.); fanw92@nwpu.edu.cn (W.F.); zongheng001@e.ntu.edu.sg (Z.F.); 2 School of Mechanical Science and Engineering, Northeast Petroleum University, Daqing 163318, China; 3 School of Mechatronics Engineering, Harbin Institute of Technology, Harbin 150001, China; 2023111865@stu.hit.edu.cn; 4 Zhengzhou Research Institute, Harbin Institute of Technology, Zhengzhou 450000, China

**Keywords:** additive manufacturing, petrochemical, remanufacturing, mechanical property

## Abstract

The growing consumption of oil and gas resources and the increasing difficulty of extraction have created major challenges for traditional manufacturing and maintenance, particularly in the timely supply of critical components, customized production, and complex structure fabrication. Additive manufacturing (AM) technology, with its high design freedom, precision, and rapid prototyping, provides new approaches to address these issues. However, systematic reviews of related efforts are scarce. This paper reviews the applications and progress of metal and non-metal AM technologies in oil and gas extraction and gathering engineering, focusing on the just-in-time (JIT) manufacturing of failed components, the manufacturing and repair of specialized equipment and tools for oil and gas extraction and gathering, and artificial core and reservoir geological modeling fabrication. AM applications in this field remain exploratory and face challenges with regard to their standards, supply chains, materials, and processes. Future research should emphasize developing materials and processes for extreme conditions, optimizing process parameters, establishing standards and traceability systems, and integrating AM with digital design and reverse engineering to support efficient, safe, and sustainable industry development. This work aims to provide a reference for advancing AM research and engineering applications in the oil and gas sector.

## 1. Introduction

Since Chuck Hull invented additive manufacturing (AM) in the 1980s, the technology has advanced significantly over the past four decades [1]. Several branches have emerged from the original photopolymerization and powder sintering technologies, distinguished by their different energy sources, materials, and processes. Despite these variations, the core principle remains the same: the layer-by-layer deposition of materials to create three-dimensional objects [2,3]. AM offers several advantages, including high precision, design flexibility, and the ability to produce multi-material or functionally graded materials (FGMs). These features are particularly beneficial for producing large, thin-walled parts and specialized components, enabling rapid fabrication without the need for molds. AM has been widely applied in industries such as aerospace [4], biomedical [5], and automotive [6]; however, in the petrochemical sector [7], AM technology research and application are still at an early stage. In 2017, Lloyd’s Register first certified AM titanium parts for the oil and gas sector and, together with the Welding Institute, issued the “Guidance Notes for Additive Manufacturing of Metallic Parts” [8]; later, the American Petroleum Institute formulated application standards (API 20S [9] and API 20T [10]) specifying requirements for AM component processes, production, identification, and documentation. Additionally, ASTM International, ISO, ASME, and DNV have all successively issued related standards [11]. At present, as global oil and gas resource consumption increases and extraction becomes more difficult, AM, as a key technology, shows clear advantages in the just-in-time (JIT) manufacturing of failed parts, manufacturing and repairing special equipment/tools, and fabricating artificial cores and reservoir geological models.

1. The JIT manufacturing of failed parts: In some extreme climates and offshore environments, it is crucial to maintain an adequate stock of critical spare parts to avoid long downtimes and production disruptions due to the unavailability of replacement parts. This need calls for careful inventory management. Wear-prone parts, such as burner plugs, choke cage valves [12], tubing hanger protectors [13], and others, typically need to be remanufactured and shipped after failure. The use of AM allows spare parts to be manufactured near the operational site. This approach supports the JIT manufacturing of failed parts, minimizing inventory and transportation costs, reducing downtime, and ensuring continuous production.
2. The manufacturing and repair of specialized equipment and tools for oil and gas extraction and gathering: The geometric structure of oil and gas extraction equipment components is complex, and the operating environment is harsh. Components used in oil and gas gathering pipelines (onshore and subsea) [14,15], oxygen–hydrogen micromixer [16], high-pressure pipeline components [17,18], valves [19], electrical submersible pumps (ESPs) [20], hydrocyclones [21], and small downhole tools [22] are subjected to complex chemical and physical stresses. These components must also meet stringent standards for service life, corrosion resistance, and reliability. AM allows for the manufacturing or repair of these components with a high degree of flexibility and precision. Additionally, applying multi-metal AM for repairs supports the “repair as strengthening” approach, which helps extend the equipment’s service life.
3. Artificial core and reservoir geological modeling fabrication: During the development of complex and tight oil reservoirs, traditional technologies face difficulties in characterizing the internal structure of the reservoir and the microscopic pore and throat features of the core. The integration of CT, 3D scanning, and AM enables the effective and intuitive characterization of the internal structure of complex reservoirs, as well as the micro- and nanoscale features of cores with high precision. This provides an effective and intuitive method for creating reservoir geological models [23] and fabricating artificial cores [24].

The total market value of the oil and gas AM market is expected to reach USD 31 billion by 2030, representing significant economic value [25]. Although AM technology has been applied across different stages of oil and gas extraction and gathering engineering with promising results, evaluations of these applications have not yet been reported. To address this gap, we conducted a comprehensive review summarizing the current state of the research and application of AM technology in the JIT manufacturing of failed parts, the manufacturing and repair of specialized equipment and tools for oil and gas extraction and gathering, and artificial core and reservoir geological modeling fabrication. We analyzed the challenges and future trends in applying AM technology to oil and gas extraction and gathering engineering, aiming to provide valuable references and insights to support further research and practical applications in this field.

### 1.1. Additive Manufacturing

According to the ASTM standard F2792 [26], AM technologies are categorized into seven distinct groups: binder jetting (BJ), directed energy deposition (DED), material extrusion (ME), material jetting (MJ), powder bed fusion (PBF), sheet lamination (SL), and vat polymerization (VP). Table 1 shows the materials that can be processed by the various technologies, along with their direct or potential applications in the petrochemical sector.

The basic principle of material extrusion (ME) technology is to heat thermoplastic filaments and extrude them through a nozzle, relying on intermolecular diffusion to achieve interlayer bonding. The equipment is portable and cost-effective, but it has weak interlayer bonding, significant mechanical property anisotropy, and limited precision. It is mainly applied to non-load-bearing components, such as pipe insulation covers and cable clamps for on-site auxiliary use. Material jetting (MJ) uses a nozzle to spray photopolymer resin droplets, which are instantly cured by an ultraviolet light source to achieve voxel-level control. While it offers high precision, the material strength is low, making it suitable for producing prototypes with complex geometries or high surface quality requirements. Sheet lamination (SL) involves cutting and bonding or welding metal foils or composite material sheets layer by layer to form the final product. It has very low costs and no thermal deformation, but limited interlayer strength, so it can be used for pipe insulation shell prototypes and tank concept models.

**Figure 1:**
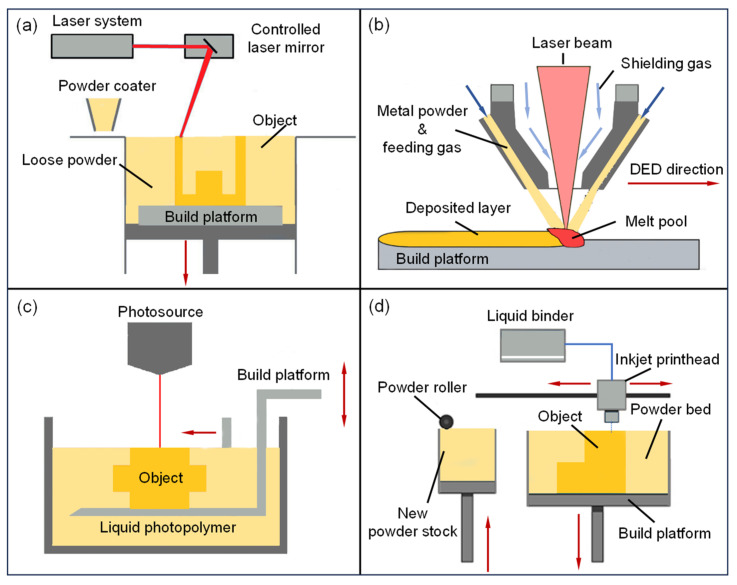
Schematic diagrams of technologies commonly used in AM for oil and gas extraction and gathering engineering: (**a**) powder bed fusion (PBF), (**b**) directed energy deposition (DED), (**c**) vat polymerization (VP), (**d**) binder jetting (BJ) [27,28,30,31].

The above three AM technologies have limited application scenarios in oil and gas extraction and gathering engineering due to their inherent technical and material characteristics. They are suitable for non-load-bearing components, auxiliary parts, and rapid prototyping, but specific applications have not been clearly reported. Powder bed fusion (PBF), directed energy deposition (DED), vat photopolymerization (VP), and binder jetting (BJ) are more common in oil and gas extraction and gathering engineering. The basic principles of these four AM technologies are shown in Figure 1. They will be described in detail below.

### 1.2. Powder Bed Fusion (PBF)

The basic principle of PBF technology is shown in Figure 1a. Using rollers or scrapers, metal powder is evenly spread to form a powder bed. An energy source selectively melts the designated areas, and the final part is formed through layer-by-layer deposition. This technique achieves a high dimensional accuracy and a low surface roughness, making it suitable for forming a wide range of metals and metal matrix composites. The powder bed also provides support, reducing reliance on auxiliary support structures, and is particularly suitable for manufacturing parts with complex geometries or overhanging structures. At present, PBF is used to fabricate key components, such as thermal insulation housings for logging tools, corrosion-resistant ceramic valve cores for refineries, valve and pump assemblies in gathering and transportation pipeline networks, and nylon filters for oil–water separators. The maximum forming dimensions have reached 1258 mm × 1258 mm × 2000 mm [33]. Based on the type of energy source, PBF technologies can be classified into LPBF and EBM. Based on whether the powder is fully melted or not, they can be classified into SLM and SLS [27]. Among these, the SLM process fully melts the powder during forming, resulting in a higher component density (>97%), and is widely used for manufacturing metal parts with high mechanical performance requirements. SLS uses functional binders (such as thermoplastic binders, chemical binders, hardeners, surfactants, etc.) to bond the powder particles together, followed by post-processing to remove impurities, achieving a density of 92−97%. SLS is capable of processing a wider range of materials.

In the PBF process, the chemical composition selection of composite powder materials is crucial for forming quality and service performance, especially in oil and gas extraction and gathering engineering, where components often work under high-temperature, high-pressure, and strongly corrosive conditions. Generally, metal matrix materials are mainly selected from Ni-based, Co-based, or Fe-based alloys with excellent metallurgical properties. To improve wear resistance, hard phase particles, such as WC and TiC, are commonly introduced into the matrix [34,35], forming metal matrix composites that enhance the wear resistance of easily worn components, like valve cores and drill bits. The composition ratio of the composite powder should take into account the service environment, functional requirements, and compatibility with the forming process. In media containing sulfur, chlorine, and other strongly corrosive substances, adding Cr and Mo to the matrix can enhance corrosion resistance [36]. To ensure good flowability and uniform powder spreading while preventing element segregation, it is also important to reasonably control powder particle size distribution and element proportions. In addition, process parameters, such as laser power, scanning speed, and layer thickness, have a significant impact on the material’s chemical composition and the properties of the final product. These parameters directly determine the melt pool’s temperature field and solidification conditions, thereby affecting the grain size and phase distribution. An excessively high energy density may cause the evaporation of low-boiling-point elements and lead to defects like porosity. The dilution rate also changes with the process parameters, influencing the final chemical composition and mechanical properties.

Numerous reviews have already addressed key issues in PBF. J. F. et al. [37] summarized common defects in PBF technology, categorizing them by scale into geometric defects, surface integrity defects, and microstructural defects, and reviewed relevant control methods, which are crucial for the production of high-precision, specialized parts in the petrochemical industry. Y.Q. Zhou et al. [38] reviewed the relationship between micro-defects, grain shape/size, process parameters, and the corrosion resistance of PBF stainless steel, providing insights for optimizing process parameters in corrosion-resistant, high-pressure vessels. Zheng L. et al. [39] provided a detailed review of the causes of spatter in PBF technology, in situ detection methods, their impact on the process and final product, and potential improvement methods, contributing to the advancement of industry applications in the additive manufacturing and repair of oil equipment/tools. In the field of multi-material PBF, Chao W. et al. [40] reviewed the deposition mechanisms, melt pool behavior, and process characteristics of different material combinations in multi-material PBF. In particular, L.M. Yao et al. [27,41] further investigated issues such as dissimilar metal compatibility, element segregation, and grain growth mechanisms in multi-metal PBF, finding that multi-metal additive manufacturing enables the additive repair and remanufacturing of petrochemical components. Compared to single-metal PBF, multi-metal PBF is better suited for applications in drilling equipment, heat exchangers, and piston pumps due to its ability to form FGMs. The recent research on multi-metal components produced by PBF technology is summarized in Table 2.

### 1.3. Directed Energy Deposition (DED)

The basic principle of DED is illustrated in Figure 1b. It uses a high-energy beam (laser, electron beam, or arc) to create a localized molten pool on the substrate surface, while simultaneously feeding a metal wire or powder into the molten pool in a coaxial or off-axis manner. The material melts at high temperatures and rapidly solidifies, forming parts layer by layer. DED technology offers a high deposition efficiency and large forming dimensions. It is typically combined with high-precision industrial robots or gantry systems to control the nozzle path, and it is widely applied to the manufacturing of large, complex components, the repair of in-service parts, and the custom fabrication of multi-material gradient structures [52]. Compared to PBF technology, DED depends more heavily on support structures for overhanging features, leading to a relatively lower surface quality and forming precision, and usually requires secondary machining [53]. DED technology can be categorized into two types based on the material form: wire-based and powder-based. Technologies using metal wire as a raw material include laser wire welding additive manufacturing (LWWAM) and arc wire additive manufacturing (WAAM), which offer high deposition rates and material utilization, making them suitable for the rapid production of medium- and large-sized components. In contrast, Laser-Based Directed Energy Deposition (LDED) technology uses an inert gas to coaxially feed powder into the molten pool, achieving a higher forming precision and being suitable for high-precision complex component manufacturing and in situ repair applications [54]. Among these, the coaxial powder-feeding method offers significant advantages in controlling the melt pool morphology and the heat-affected zone, with a stable forming process and uniform microstructure (a surface roughness as low as 5 μm) [55].

During the DED process, the substrate and filler material form a molten pool together under a high-energy density input. The molten pool experiences intense convection driven by microscopic forces, such as buoyancy, gravity, recoil pressure, and Marangoni force, leading to element diffusion, alloying reactions, and metallurgical fusion and forming a continuous, dense, and strongly bonded interface [56]. Concurrently, the solidification of the molten pool may generate intermetallic compounds, carbides, and other phases, ultimately affecting mechanical properties, such as fatigue strength, hardness, and wear resistance. By designing the composition and optimizing the process parameters, the solidification process of the molten pool can be partially controlled. Process parameters, such as the laser power, scanning speed, and powder feed rate, directly determine the thermal input and cooling rate of the molten pool, significantly influencing the chemical composition’s uniformity and the microstructural morphology of the formed part [57]. A high-energy density input may cause the evaporation or segregation of low-boiling-point elements, leading to compositional inhomogeneity; changes in the cooling rate can alter the grain size and the secondary phase distribution, thereby affecting mechanical properties. Matching process parameters that are suitable for the material not only improves the forming quality but also significantly enhances the mechanical properties of the deposited layer, meeting the stringent operational requirements of oil and gas extraction and gathering engineering. DED technology is widely applied in the repair and manufacturing of critical components, such as high-pressure valves and manifolds. The reasonable matching of materials and process parameters can effectively optimize melt pool wettability, solidification microstructure, and defect sensitivity, thereby improving the comprehensive mechanical properties of the deposited layer. Recently, review articles on DED have primarily focused on improving mechanical properties. Kun et al. [58] summarized the mechanisms of micro-defects evolution during the deposition of steel using DED and proposed methods for improvement. Chao Wei [59] highlighted the advantages of DED in enhancing the interface properties of FGMs. In recent years, methods such as ultrasonic-assisted [60] and subtractive [61] have also been used to optimize process flows, reduce residual stress, and enhance mechanical properties, demonstrating the broad application prospects of DED in petroleum equipment, such as deep-sea pipelines, ultra-deep well drill pipes, and corrosion-resistant repair layers.

### 1.4. Vat Polymerization (VP) and Binder Jetting (BJ)

The mechanical performance requirements for equipment and tools used in oil and gas extraction and gathering engineering are high, with strict safety standards. As a result, the application of metal AM is more widespread. However, non-metal AM also plays a significant role. VP can be used for the fabrication of artificial cores and reservoir geological models for displacement experiments, while BJ is commonly used for the fabrication of artificial cores and molds for impellers and drill bits.

The basic principle of VP is shown in Figure 1c. An ultraviolet light source (either a point source or a surface projector) selectively illuminates the resin tank, causing the liquid, photosensitive resin inside to undergo a photopolymerization reaction under UV light and instantly cure in the illuminated area. This process is repeated layer by layer to create a three-dimensional object. This technology provides the high precision and low surface roughness of the molded parts, but the material is brittle and has poor temperature resistance. Based on the light source, it can be further classified into SLA, DLP, and CDLP/CLIP [62]. Among these, SLA offers the highest resolution, with a minimum feature size of 10 μm [63], while DLP and CDLP/CLIP use digital light sources that enable the direct projection of single-layer images, achieving a maximum build speed of up to 1200 mm/h [64]. From a mechanical performance perspective, the build platforms of SLA and DLP move layer by layer during the printing process, resulting in a staircase effect, which leads to anisotropy in mechanical properties. In contrast, the build platform in CDLP/CLIP moves continuously, significantly reducing the anisotropy [65]. Recently, review articles in the field of VP have mainly focused on material development and performance optimization [66], multi-material and multifunctional printing [31], process parameter optimization, and improvements in mechanical properties [67].

The basic principle of BJ is shown in Figure 1d. Powder is applied layer by layer using a scraper or roller, and the inkjet head selectively sprays a binder according to the slice model, locally curing the powder to form a green part. After removing loose powder, the green part is densified through high-temperature sintering in a protective atmosphere furnace. This technology does not require additional support, and in theory, all powders that can be bonded can be shaped [32]. Since the forming process does not involve high-temperature heat sources, it avoids the deformation and cracking caused by thermal stress. However, during post-processing, dimensional shrinkage may occur, and mechanical properties may be relatively weak [68]. Recently, Mostafaei, A. [69] discussed the effects of powder characteristics, process parameters, and post-processing on the porosity and mechanical properties of BJ-formed products. Guanxiong, M. et al. [70] provided a detailed analysis of how the quality of the powder bed is influenced by the powder spreading equipment, process parameters, and powder characteristics during the powder bed setup and summarized the micro-forces that may lead to powder agglomeration.

## 2. Application of AM in Oil and Gas Extraction and Gathering Engineering

### 2.1. JIT Manufacturing and Application of Failed Components in Oil and Gas Extraction Site

In recent years, the application of AM in the oil and gas industry has gradually expanded, with significant cost differences compared to traditional processes, such as casting and forging. AM can reduce inventory, shorten delivery cycles, and customize complex structures, but the direct manufacturing cost per part is usually higher than that of traditional processes. According to research by Mecheter et al., the manufacturing cost comparison between typical AM parts and traditional process parts for SS316L material is shown in Table 3 [71].

Oil and gas extraction sites are typically located in remote areas or offshore platforms. To ensure continuous production, it is essential to maintain a critical spare parts warehouse. These components require high standards for equipment and technical personnel during the production process, relying on the original manufacturers or specialized suppliers. Due to lengthy logistics, delivery to the operational site can take a significant amount of time. AM enables the precise and rapid production of critical spare parts, helping to avoid prolonged downtime that could lead to substantial economic losses [72]. Compared to traditional manufacturing methods, which require extensive production lines, AM equipment requires less space and fewer personnel, allowing deployment in small, local factories to produce parts on demand [73]. This supports digital and localized production, reducing storage and labor costs. With advancements in reverse engineering technology, engineers can obtain CAD models of critical spare parts [74], enabling them to pre-establish process parameters and create virtual inventories, further alleviating inventory pressures. Some well-known oil companies have actively explored this approach, yielding positive results.

**Figure 2:**
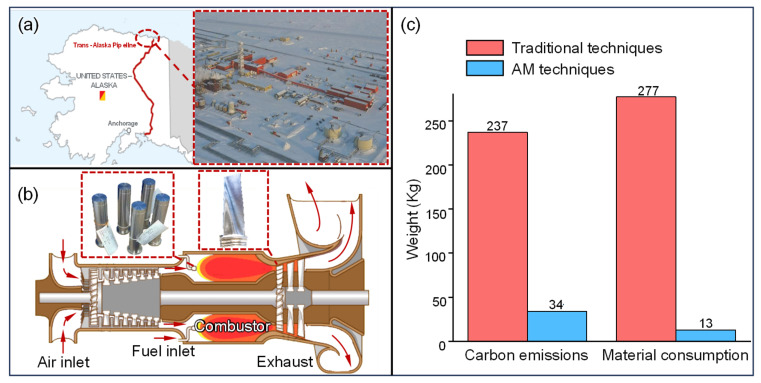
The application of AM in the Kuparuk oilfield and offshore platforms. (**a**) The geographical location of the Kuparuk oilfield. (**b**) Gas turbines for the oilfield’s power supply: the working principle, burner plugs, and blades. (**c**) A comparison of the carbon emissions and material consumption between AM and conventional technologies in the production of tubing hanger protectors [77].

ConocoPhillips’ Kuparuk oilfield, located on Alaska’s North Slope [Figure 2a], faces significant challenges in the supply of critical spare parts due to its remote location and harsh environmental conditions. Parts that are no longer in production can only be sourced from the nearest city, Fairbanks, which is approximately 400 km away. For parts still in production, direct orders from the original manufacturer can take nearly a year before they are ready for assembly. To address this issue, at the end of 2023, ConocoPhillips formed a team to provide AM solutions. To date, the team has successfully produced AM burner plugs for gas turbines [Figure 2b]. These gas turbines compress the associated natural gas produced during extraction and reinject it into the reservoir to enhance oil recovery. In the process, they also generate electricity to power production equipment and infrastructure [75]. The burner plug is a wear-prone component of the gas turbine. However, the original manufacturer no longer produces this model, making part replacement extremely difficult. By leveraging AM, ConocoPhillips can now produce the plugs within 14 to 21 days, significantly reducing the lead time. In practice, the mechanical performance of the AM plugs has been demonstrated to be superior [12]. Oak Ridge National Laboratory in the U.S. completed the production of a large turbine blade within two weeks using WAAM technology [Figure 2b], greatly reducing the delivery time compared to traditional casting methods, which typically take 7 to 8 months [76].

The floating production storage and offloading system (FPSO), equipped with comprehensive production facilities, storage capacities, and offloading operations, is primarily used for offshore oil and gas extraction, processing, storage, and offloading. It features high flexibility and a low environmental impact but comes with significant maintenance demands [78]. Recently, AM equipment was deployed on the FPSO SKARV to ensure the supply of critical spare parts during maintenance periods [79]. The limited storage space on an FPSO is not conducive to maintaining an inventory of critical spare parts. With AM, engineers can establish a digital inventory and maintain only a small stock of raw materials [80]. Of course, AM is not suitable for all parts. The Brazilian oil service company Ocyan has experimented with the DigiPART software platform, analyzing the feasibility of AM for 17,000 inventory parts and finding that at least 11% of the parts are suitable for production using AM [81]. The Norwegian company Aker BP used SLM technology to manufacture tubing hanger protectors [13], which protect hydraulic and electrical control elements during pipeline installation. Compared to traditional manufacturing methods, AM can significantly reduce material usage and CO_2_ emissions [Figure 2c].

However, in the aforementioned application cases, there has been no further evaluation of the corrosion resistance of AM components used in marine environments. Recently, Behzad A et al. [7] proposed a risk assessment method for offshore platform AM components, providing a reference for the development of certification and inspection schemes. Several studies have studied the corrosion resistance of AM steels in marine environments, as shown in Table 4. Compared to traditional manufacturing processes, AM allows for better control over microstructure and surface oxide film formation during the fabrication process and enables material composition optimization based on actual service conditions to enhance corrosion resistance and other mechanical properties. Regarding other corrosion-resistant alloys and coatings used in marine environments, C. Arcos [82] analyzed the corrosion resistance of nickel aluminum bronze (NAB) with different heat treatments from both microstructural and electrochemical perspectives. Clara Linder et al. [83] enhanced the corrosion resistance of an Al-Mn-Cr-Zr alloy using AM technology and tested its corrosion resistance in a natural marine environment. The modified Al-Mn-Cr-Zr alloy was able to form a large-area surface passivation film, which significantly improved its pitting resistance. C.J. Todaro et al. [84] explored the feasibility of LPBF-formed IN725, providing appropriate process parameters with a relative density of 99.5% (scan speed *v* = 700 mm/s, hatch distance *h* = 60 μm). After heat treatment, the tensile properties of the AM components were nearly identical to those of forged parts and showed excellent corrosion resistance, offering a reference for forming IN725 components for use in marine environments. V. Rajkumar et al. [85] used WAAM to produce IN825 components and analyzed their corrosion resistance (annual corrosion rate of 0.59–0.7 mpy). Jiaqi Li et al. [86] systematically studied the corrosion resistance of a Ti-8.5Cu alloy produced using DED, shedding light on the corrosion mechanisms in sterile and non-sterile seawater environments.

### 2.2. Manufacturing and Repair of Specialized Equipment/Tools for Oil and Gas Extraction and Gathering

#### 2.2.1. Manufacturing and Maintenance of Oil and Gas Gathering Pipelines and Components

Oil and gas gathering pipelines must operate under extreme conditions, including high-pressure and corrosive environments. Additionally, some buried pipelines must withstand soil pressure, making them vulnerable to corrosion, cracking, mechanical damage, and even perforation or misalignment, which can disrupt continuous production and pose a threat to human safety [93]. Traditional pipeline repair methods include fixture-based repairs, pipeline welding, and composite material patching, but these methods are often expensive, time-consuming, and lack precision. In contrast, additive manufacturing (AM) provides a more efficient, precise, and flexible solution. In November 2022, Shell unveiled a leak repair clamp [94] produced using WAAM, which can be manufactured in just 3 to 5 days, significantly reducing the production time compared to traditional leak repair clamps. This advantage is particularly evident in the production of complex pipeline leak clamps, and its pressure resistance exceeds the design pressure by five times. Furthermore, ConocoPhillips’ choke cage valve [12] and Shell’s oxygen–hydrogen micromixer [16] further demonstrate the strong adaptability of AM in the production of specialized pipeline components. Currently, DED is the leading technology for chemical pipeline maintenance and repair [95]. By adjusting process parameters and repair materials, it has been shown to significantly improve the mechanical properties at damaged locations [96,97]. CAD model characterization is a key issue in pipeline repair. Traditionally, repair solutions are based on comparing pre- and post-damage models, a reliable and effective approach that requires standard models for reference [98]. However, with advancements in artificial intelligence and machine vision, researchers have developed characterization methods that do not require standard models, greatly reducing the time needed to construct models. Hamdan M.A. et al. [14] proposed an automatic path planning method for scanning, which successfully repaired typical pipeline defects. This automatic path planning method reduces path planning time by approximately 75%, while ensuring repair effectiveness, and is significant for the rapid and high-precision repair of pipelines.

**Figure 3:**
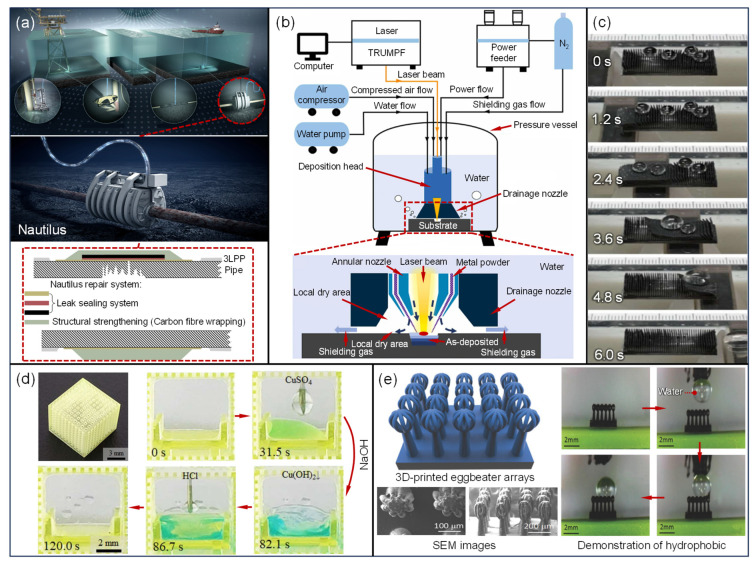
(**a**) Kongsberg Ferrotech’s digital pipeline maintenance system and Nautilus robot repair platform. (**b**) A schematic of the underwater direct metal deposition (UDMD) test device for subsea oil pipelines. (**c**) The droplet cleaning process for subsea pipeline wall surfaces based on micro-patterned wall arrays. (**d**) Research on the bio-inspired, self-cleaning structure of microstructures in hornwort for subsea pipeline corrosion protection. (**e**) Research on the anti-corrosion properties of subsea pipelines based on the hydrophobic characteristics of eggbeater-inspired structures [107,108,109,110].

Subsea pipelines play a crucial role in the gathering of offshore oil and gas. However, due to the harsh service environment, they are vulnerable to damage from corrosion, fatigue, and mechanical stresses, leading to pipeline deformation, wall thinning, and even leaks [99,100]. Traditional subsea pipeline repairs rely on divers and remotely operated vehicles (ROVs), which are time-consuming, pose safety risks, and face challenges when performing maintenance in deep-sea environments. To address this, Norway’s Kongsberg Ferrotech introduced the Nautilus underwater pipeline repair robot with automatic recognition capabilities [Figure 3a]. Nautilus can autonomously clamp the pipeline, drain seawater, locate defects, apply repair fluids and anti-corrosion coatings, and wrap the repair materials [101]. This innovation significantly reduces the need for underwater personnel for pipeline inspection and maintenance, offering promising potential for deep-sea pipeline maintenance. However, Nautilus currently only performs patch repairs and lacks additive manufacturing (AM) capabilities. Shell has partnered with Kongsberg Ferrotech to integrate AM functions into Nautilus, expanding the device’s range of applications [15]. The basic principles of underwater AM are shown in Figure 3b. Recently, Guifang Sun’s team at Southeast University summarized the differences in melt pool convection, conduction, and metallurgical dynamics between onshore and subsea directed energy deposition (DED) processes [102] and investigated the fatigue behavior of underwater DED [103], the solidification process of different metal melt pools under deep-water, high-pressure conditions [104], the relationship between microstructures and mechanical properties [105], and the optimization of corrosion resistance [106]. At present, underwater AM mainly involves single metal/alloy materials and holds significant potential for in situ subsea pipeline repair, although this method does not fundamentally slow down pipeline degradation or damage. Some researchers, inspired by biomimetic principles, are attempting to use superhydrophobic structures to prevent direct contact between seawater and pipelines, thus reducing subsea pipeline damage. Sangyeun Park and colleagues [107] combined biomimetic principles, magnetoresponsive materials, and AM to print micro-patterned wall arrays, which exhibited superhydrophobic properties under certain conditions [Figure 3c]. Inspired by the microstructures of hornwort, Mingzhu X [108] designed a porous biomimetic structure with a hydrophilic outer surface and a hydrophobic inner surface, demonstrating excellent unidirectional fluid performance for rapid solid–liquid separation [Figure 3d]. Yang [109], inspired by Salvinia molesta, used a resin composite material infused with multiwalled carbon nanotubes to print eggbeater artificial hairs with a hydrophobic performance using hydrophilic materials [Figure 3e]. These developments highlight the potential applications of AM biomimetic structures in subsea pipeline corrosion protection and oil–water separation from a design perspective. However, due to material durability limitations and the extreme deep-water pressure and temperature, no mature technologies or application cases have been reported yet for either the AM in situ repair of subsea pipelines or AM biomimetic coatings.

**Figure 4:**
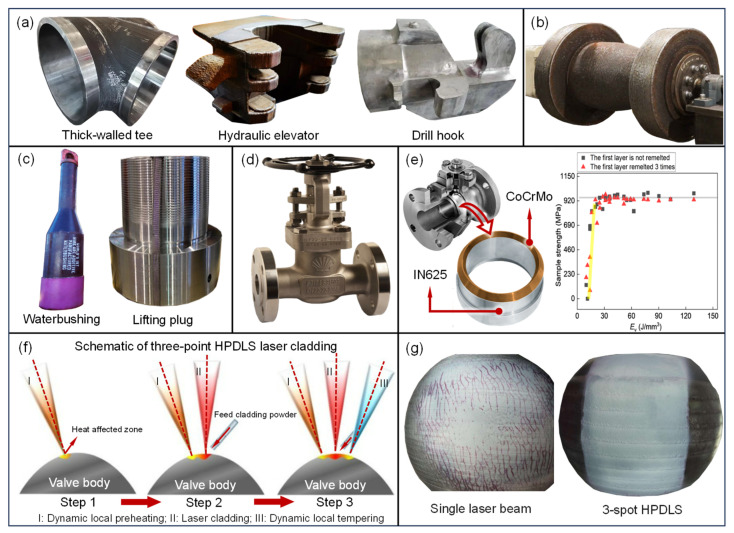
Application of AM in high-pressure pipeline components and valves: (**a**) large AM components by China Petroleum Engineering Materials Research Institute, (**b**) pressure pipeline components ordered from AML3D, (**c**) AM waterbushing and lifting plugs produced by Vallourec, (**d**) gate valve printed by Shell and Bonney Forge, (**e**) ball valve core reinforcement in chemical pipelines and tensile strength of dissimilar metal interfaces at varying laser energy densities, (**f**) schematic diagram of principle of high-power diode laser source process for valve core repair, (**g**) comparison of liquid dye penetrant test for valve core repair using traditional laser cladding and high-power diode laser source [133].

In high-pressure pipeline components, the China Petroleum Engineering Materials Research Institute [17] became the first to use WAAM to produce large-diameter, thick-walled tees for low-temperature oil and gas gathering pipelines, with the pressure resistance reaching 57.5 MPa. As a critical component of these pipelines, the mechanical properties and load-bearing capacity of these tees directly affect the integrity and gathering efficiency of the pipelines. Compared to the traditional hot drawing process, AM can effectively improve the anisotropy of mechanical properties, preventing brittle instability and cracking [111]. The team also explored printing hydraulic elevators and drilling hooks [Figure 4a]. ExxonMobil Corporation ordered a high-pressure pipeline vessel (weighing 940 kg, 850 mm long, 450 mm in diameter, and with a wall thickness of 41 mm) from Australian (Nth Plympton) AM company AML3D for USD 190K. Produced using directed energy deposition (DED) within 12 weeks, this vessel reduced the delivery time by over 50% [Figure 4b]. The company claims that their DED technology can reduce manufacturing costs for large aluminum, titanium, nickel, and steel components by 70% compared to traditional technologies and reduce production waste by 85% [18]. In February 2021, Vallourec utilized WAAM to produce a waterbushing (1.2 m long, weighing 220 kg) designed to prevent oil and gas leakage. The waterbushing has undergone more than 150 rigorous tests and is currently operational at the Elgin–Franklin field in the North Sea. That same year, Vallourec also produced lifting plugs with load capacities of hundreds of tons, designed for workover operations off the coast of Australia [Figure 4c] [112,113]. High-pressure pipeline components are highly sensitive to micro-defects and residual stress, which can be addressed through in situ recrystallization for stress relief, the precise control of microstructure morphology, and temperature gradient management to enhance mechanical properties. The relevant results are summarized in Table 5. Additionally, new materials can be developed through alloy design. Recently, a team led by Professor Qian Ma from RMIT University [114] developed a strong and ductile α-β titanium–iron–oxygen alloy by combining alloy design with AM, using industrial waste (such as sponge titanium or sponge titanium–oxygen–iron) as raw materials. By controlling process parameters, the team achieved the optimal coordination of oxygen and iron elements, resolving the issue of “oxygen embrittlement”. This alloy shows significant potential for use in high-pressure pipeline components, high-pressure storage tanks, and offshore oil equipment. However, considering the dynamic loads caused by fluid fluctuations, mechanical vibrations, and thermal shocks during valve operations and pump activities in oil and gas gathering, it is critical to evaluate the dynamic response of mechanical properties and fatigue to further ensure the service reliability of AM components.

Valves are essential in oil and gas gathering, serving functions such as shutoff, regulation, and pressure stabilization. The gate valve produced through a collaboration between Shell and Bonney Forge has been certified by DNV and is currently in service at the Rotterdam plant [Figure 4d] [19]. However, further evaluation reports have yet to be released. In practice, valves are subjected to complex chemical and physical stresses from the conveyed medium and the service environment. To ensure reliability, multiple components are reinforced with coatings [132]. Traditional methods, like welding and cold/hot spraying, create thin layers with weak interfacial bonding that are prone to cracking, rendering them unsuitable for long-term use. To extend the valve’s lifespan and reduce downtime, AM can be used to enhance its mechanical performance. Hoo S. Z., L. M. Yao, and colleagues [133,134] applied PBF to deposit a 10 mm corrosion-resistant coating (CoCrMo) on commonly used materials for valve cores (SS316L) and investigated the relationship between the laser volumetric energy density and the interfacial tensile strength and hardness [Figure 4e]. As the laser volumetric energy density increases, the tensile strength and hardness of the interface initially increase and then decrease. When the laser volumetric energy density is between 35 J/mm^3^ and 75 J/mm^3^, the average tensile strength is approximately 903 MPa, and the average interface hardness is approximately 382 HV. However, this study only demonstrated the feasibility from a materials perspective. Due to the complexity of valve structures, directed energy deposition (DED) is more suitable for reinforcing valves. Considering the structural complexity and the coating thickness, process adjustments are necessary to minimize thermal stress and prevent cracking. Xingchen L. et al. [135] proposed a high-power diode laser process that utilizes triple laser sources for dynamic local preheating, laser cladding, and dynamic local tempering to adjust thermal stress in real time and prevent cracking, which was validated on valve cores [Figure 4f,g]. Shengguan Q et al. [136] conducted wear tests using a friction tester to evaluate the wear resistance of reinforced valves. The mass loss of the reinforced valves was only half that of conventional valves, which is significant for assessing their wear resistance in real-world service environments.

#### 2.2.2. Manufacturing and Repair of Downhole Tools/Equipment

**Figure 5:**
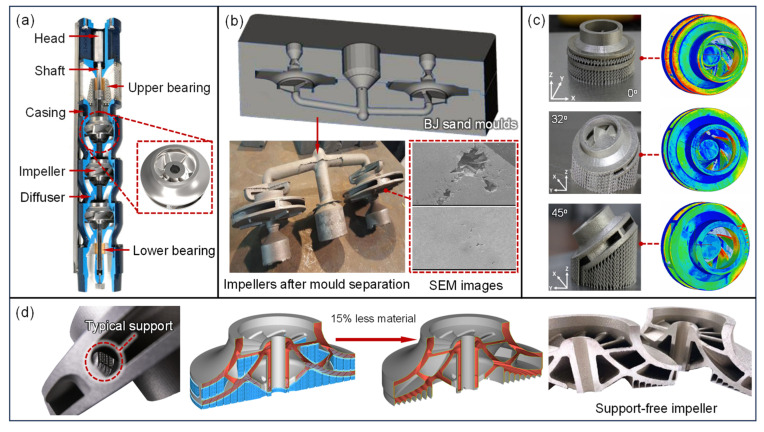
Application of AM in drilling equipment: (**a**) schematic of ESP structure, (**b**) ESP impeller produced with BJ assistance and SEM images of metal shrinkage defects during cooling, (**c**) fabrication of SLM impellers at various inclination angles and assessment of geometric accuracy, (**d**) support-free impeller produced by EOS [20,139,141].

ESP is a crucial piece of equipment in oil and gas extraction [Figure 5a]. The main failure modes of the impeller, influenced by the transporting medium, periodic stresses, and temperature, include blade wear, corrosive perforation, cavitation damage, and fatigue cracks [137,138]. The challenges in impeller manufacturing lie in the high-precision fabrication of blade shapes, especially in dealing with non-developable ruled surfaces, variable wall thickness non-ruled surfaces, and multi-point fitted surfaces with varying wall thicknesses. Multi-axis machining centers are necessary to ensure manufacturing accuracy. Most ESP impellers are closed designs, necessitating the separate machining of the blades and front and rear cover plates, followed by assembly and welding. Felix H. et al. [139] used BJ techniques to create molds, which were then shaped through casting [Figure 5b]. While this method allows for the high-precision formation of complex impellers, it cannot avoid defects arising from metal shrinkage during the cooling process. Alexandra A. et al. [20] produced a six-blade closed impeller using LPBF technology, thoroughly evaluating the impeller’s dimensional accuracy, surface quality, and dynamic balance performance [Figure 5c]. During the direct formation of the impeller, supports are required to maintain precision, which are later removed through post-processing. The design and removal of these supports can account for up to 70% of the total production costs. Recently, the German AM company EOS proposed a support-free impeller printing solution [Figure 5d], significantly reducing the production time while saving material and post-processing costs. According to official data, this approach can achieve material savings of at least 15% and cost reductions of 35% [140]. This advancement not only provides new perspectives for the design, production, and maintenance of ESP and other turbomachinery but also opens new pathways for manufacturing specialized flow components.

**Figure 6:**
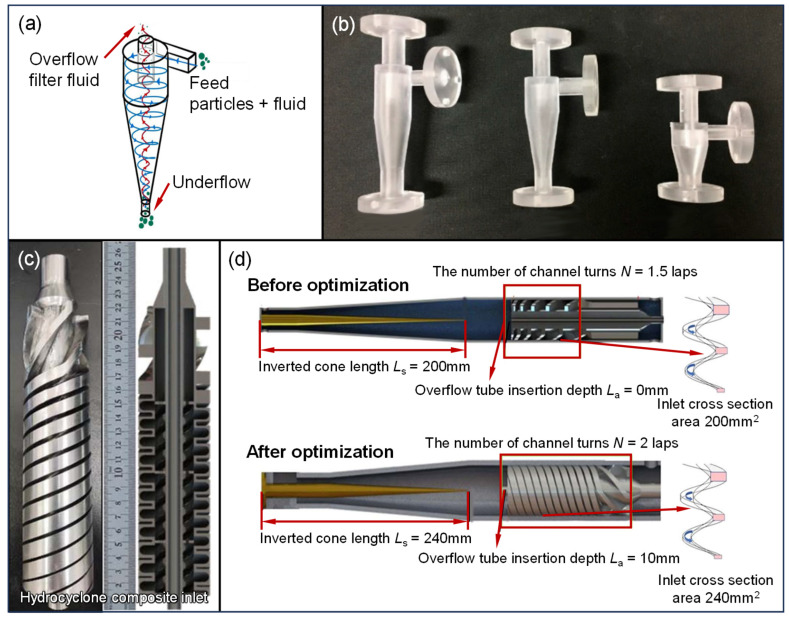
Applications of AM in hydrocyclones: (**a**) basic principles of hydrocyclones, (**b**) SLA micro-hydrocyclones, (**c**) SLM variable-pitch hydrocyclone components, (**d**) changes in hydrocyclone parameters before and after optimization [21,142,144].

As a core device in same-well injection-production technology, the hydrocyclone separator is primarily used to separate the produced fluid whilst still downhole, enhancing the recovery efficiency and reducing reinjection costs. The basic operating principle is illustrated in Figure 6a. The hydrocyclone separator consists of an upper fluid inlet and a lower conical separation chamber. To improve the separation efficiency, researchers have focused on optimizing the separator’s structure. However, traditional manufacturing methods often lead to high production costs or difficulties in fabricating components with complex flow channels or surfaces. To address these issues, some researchers have explored the use of AM technologies to fabricate critical components. Yi et al. [142] fabricated an SLA micro-hydrocyclone and measured its separation efficiency using a gravimetric method. Using a full factorial design (FFD), they identified the optimal vortex finder and outlet diameters at an outlet flow rate ratio of one, improving the separation efficiency by 1.72% [Figure 6b]. Vega-Garcia D. et al. [143] proposed a computational fluid-dynamic-based optimization method for small hydrocyclones and produced a parabolic-structured AM mini-hydrocyclone. However, these products were scaled models made of non-metallic materials, unsuitable for the complex downhole environment. Xiaojie Zhao et al. [21] utilized SLM technology to optimize the fluid inlet of a variable-pitch hydrocyclone [Figure 6c,d], increasing the initial fluid velocity and improving the separation efficiency. They also analyzed the effects of the inlet flow rate, split ratio, and oil-phase volume fraction on the separation performance. The separation efficiency of the newly designed variable-pitch hydrocyclone reached 99.38%, an improvement of 0.58% over traditional separators. These advancements provide valuable insights for the design and rapid iteration of hydrocyclone separators and offer important guidance for optimizing equipment in same-well injection-production technology.

**Figure 7:**
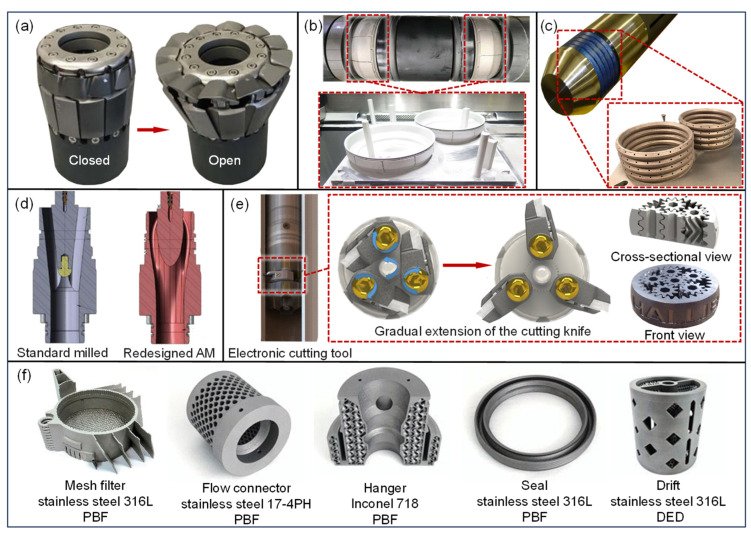
AM components for downhole tools: (**a**) Halliburton’s anti-burst rubber tube, (**b**) Baker Hughes’ high-expansion packer with AM backup ring, (**c**) DET’s metal packer for high-temperature wells, (**d**) comparison of effects of AM-based diverter optimization before and after implementation, (**e**) AM planetary gears in pipe cutting tool, (**f**) other AM components for downhole tools [145,146,147,148,150].

In downhole packers, Halliburton was the first to introduce a rubber tube anti-burst device to ensure the maximum pressure-bearing capacity of the packer sleeve [Figure 7a] [145]. To address the challenges of sealing with low-expansion-ratio packers in open-hole completions in horizontal wells, Baker Hughes developed a packer with an expansion ratio of 111%, which is at least 50% greater than that of conventional packers. This design requires a lower setting force and maintains a sealing capability of nearly 70 MPa at elevated temperatures [146,147]. The AM-fabricated backup ring [Figure 7b] features axial slots that adapt to sleeve deformation during expansion, preventing wrinkling that might compromise the seal. DET designed and manufactured high-temperature (371 °C) packers suitable for deep-well operations, using LPBF for the metal sealing components [Figure 7c] [22]. In addition to packers, AM has been widely applied in the production of specialized components for other downhole tools. Figure 7d illustrates the optimized internal flow path design of a flow diverter [148]. In conventional designs, solid particles in the diverting medium cause serious wear during extended operation, leading to an average service life of just 45% of the expected duration and as low as 25% in extreme cases. In contrast, AM-produced flow diverters achieve much longer service lives, with at least five units exceeding the expected lifespan by 65% and no significant wear detected during the most recent inspection. Figure 7e illustrates the application of AM planetary gears in a downhole pipe cutting tool [145]. These cutting tools are used to resolve certain stuck pipe issues during workover operations, functioning similarly to turning tools. Once positioned, the cutting blade extends radially to cut the casing circumferentially. The planetary gear is crucial for blade deployment; traditional planetary gears often use spur or helical teeth, which can bear lower loads and require additional structures for gear engagement, leading to reduced space efficiency. In contrast, AM planetary gears employ a herringbone tooth profile, providing smoother and more reliable transmission with improved space utilization. Beyond these applications, downhole tool components, such as mesh filters, flow connectors, and hangers, can also be efficiently manufactured using AM [Figure 7f] [149]. Some patents have referenced the application of AM-fabricated components, as shown in Table 6, but mass production has yet to be achieved.

**Figure 8:**
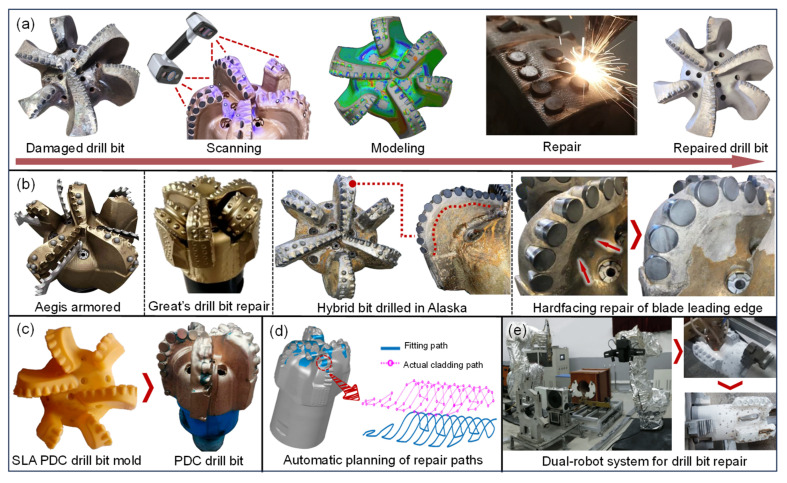
Application of AM technology in drill bit repair and remanufacturing: (**a**) workflow for drill bit repair and remanufacturing based on AM and reverse engineering, (**b**) drill bit strengthening/repair, (**c**) SLA-assisted production of PDC drill bits, (**d**) automated planning of repair paths for drill bits, (**e**) dual-robot-based automated laser cladding system for drill bit repair and remanufacturing [166,167,169,170].

PDC bits, roller cone bits, and hybrid bits are commonly used drilling tools. However, all three types of bits face challenges, such as complex manufacturing procedures, long production cycles, high costs, and high rejection rates. Enhancing the service life of drill bits and remanufacturing damaged bits are of great significance. On the one hand, it significantly reduces the maintenance frequency, facilitating continuous production; on the other hand, the cost of repairing drill bits is much lower than purchasing new ones. The drill bit repair process based on AM and reverse engineering is shown in Figure 8a. In 2019, Schlumberger developed the Aegis armor coating [Figure 8b], using EBM technology to deposit tungsten carbide material onto the cutting edge of steel drill bits. This enhanced the drill bit’s corrosion resistance and strength by 400% and 40%, respectively, extending its lifespan and reducing drilling time [163]. In 2021, Great Company used advanced remanufacturing methods, such as rigid-body overlay welding for secondary reinforcement, preform hot-melt reshaping, and diamond composite cold-press secondary embedding, to remanufacture the hybrid drill bit [Figure 8b] at just 30% of the cost of a new bit [164]. In 2023, Lingchao X et al. [165] developed a high-hardness, nickel-based graphene composite coating (Ni60A + 20% WC + 0.3% graphene) to improve drill stability in deep and ultra-deep wells. They investigated the relationship between laser power and the microhardness of the composite coating, revealing the mechanism of microstructural evolution. At a laser power of 1200 W, the microhardness reached 1031.8 HV. In March of the same year, Y. Zhang et al. [166] used EBM technology with nickel-based tungsten carbide as the reinforcing and repairing material to remanufacture PDC drill bits [Figure 8b]. This resulted in a 40% strength increase and an 80% toughness improvement. Compared to composite materials printed with commercial self-fluxing Ni-Bi alloy, the corrosion resistance improved by approximately 20%. The remanufactured drill bits have been tested in various geological environments, including the eastern U.S., northeastern U.S., Alaska, and Thailand. To improve precision and reduce production costs, Liu B et al. [167] used SLA technology to assist in the production of PDC drill bits [Figure 8c]. This approach reduced the production process from 12 steps to 6, while maintaining the machining accuracy. Field testing was conducted at well Zu203H3-4, showing significant improvements in the rock breaking efficiency (14.7% increase) and the drilling depth (7.7% increase). However, SLA-assisted mold production requires the secondary casting of molds. SLS can directly produce drill bit molds, significantly reducing the impact of human factors on the molding quality and ensuring better consistency between production and design [168]. In the fields of path planning and autonomous repair, Chuan Z et al. [169] developed a path auto-planning algorithm for repairing damaged drill bits [Figure 8d], which significantly improves the path-matching accuracy, reduces the complexity of robotic arm operations, and enhances the repair efficiency. Jian W et al. [170] developed a dual-robot automated laser cladding system [Figure 8e], which, based on hand–eye calibration, 3D scanning, and DED technology, enables the automatic scanning and repair of drill bits. A PDC drill bit repair trial was successfully conducted, but the integration and versatility of the operating system software still require further development.

### 2.3. Artificial Core and Reservoir Geological Modeling Fabrication

#### 2.3.1. Fabrication of Artificial Cores

**Figure 9:**
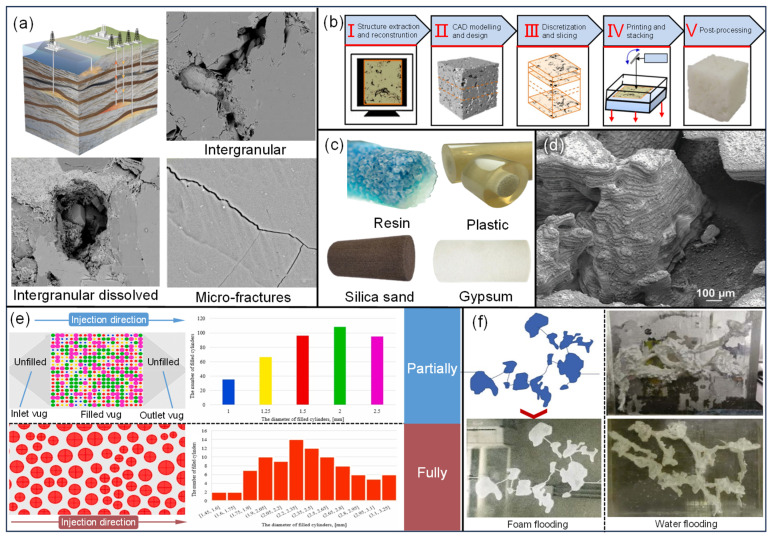
Application of AM in fabrication of artificial cores and oil–gas reservoir models: (**a**) typical pore and pore-throat structures inside oil–gas reservoir cores, (**b**) manufacturing process of artificial cores, (**c**) artificial core models made from different materials, (**d**) high-fidelity artificial cores with mineral coatings, (**e**) AM-based fractured-vuggy carbonate reservoirs model printed in collaboration between Peking University and Southern University of Science and Technology, (**f**) AM-based fractured-vuggy carbonate reservoirs model printed by China University of Petroleum [23,174,175,176].

In reservoir simulation, physical property test samples include natural and artificial cores. Natural cores effectively reflect key characteristics, such as micron-scale porosity, nanoscale throat dimensions [Figure 9a], and material wettability and adsorption properties. However, they are limited by their low availability, high costs (approximately USD 30,000/m), and susceptibility to damage during storage and testing, making reuse difficult. To overcome these limitations, artificial cores began to be fabricated around 2005. Initially, due to technological constraints and manufacturing precision, artificial cores could only represent certain physical parameters and flow behaviors. With advancements in AM and growing application demands, artificial cores have rapidly evolved to accurately simulate the properties and pore structures of natural rocks [24]. They can now model natural cores in terms of both their pore morphology and their topological properties. Artificial cores are primarily produced through BJ and micro/nano additive manufacturing (MNAM). The production process and common materials are shown in Figure 9b,c. Most reports on BJ artificial cores use natural powders. Although the current maximum resolution of BJ (approximately 100 μm) is insufficient for capturing smaller micron-sized pores and nanoscale pore throats, it offers advantages in terms of its high efficiency and large-scale production. The use of natural powders provides clear benefits for simulating the physical and chemical properties of natural core surfaces. Some researchers, seeking to characterize the mechanical properties of natural cores, have used ceramic and gypsum powders to fabricate artificial cores [171]. However, due to the limitations of these materials, the pore shapes differ significantly from those of natural cores, making them unsuitable for accurately simulating fluid flow patterns. Additionally, silica sand is another common material used for BJ artificial cores, primarily for describing macroscopic mechanical properties [172] and microstructures [173]. However, due to the limitations of both the material and the process, silica sand is not suitable for displacement experiments.

MNAM is commonly used in the production of artificial cores. MNAM refers to the creation of three-dimensional structures at the micro- and nanoscale through the layer-by-layer deposition of materials. Initially used in microelectromechanical systems (MEMS), MNAM has more recently been applied to accurately depict the microstructures of cores, ranging from several nanometers to a few micrometers [Figure 9d]. Common MNAM techniques are listed in Table 7 [24]. Projection microstereolithography (PµSL), which uses resin to produce artificial cores, is a typical example. The current minimum feature size of PµSL is 0.6 µm, making it highly suitable for characterizing the structural features and connectivity of micropores and nanopore throats [177]. Additionally, researchers can take advantage of the transparency of the resin to dynamically monitor fluid behavior within the core using digital image correlation and particle image velocimetry [178]. While AM artificial cores can accurately represent larger pore structures, limitations such as material viscosity, expansion, and other physical properties prevent the accurate depiction of low-porosity cores (e.g., tight sandstone). Specifically, challenges such as uncured material residue, difficulty in removing supports, and expansion-induced clogging can hinder the characterization of micro- and nanoscale structures, as well as fluid transport properties.

#### 2.3.2. Fabrication of Reservoir Geological Model

Many oil and gas resources have entered the mid-to-late stages of development, with reservoirs characterized by complex structures becoming an increasingly critical part of the energy mix. The precise representation of reservoir geological models is playing an increasingly prominent role in research and production. Building models based on geological, logging, and geophysical data using computer simulations is a key method in geological studies and serves as the foundation for optimizing development plans. Traditional reservoir geological descriptions, which rely on digital or manual models, face limitations when dealing with complex structures or achieving high-precision details due to material and processing constraints. AM can rapidly and accurately produce reservoir models, making it a widely used tool for visualizing displacement studies in fractured-vuggy carbonate reservoirs.

The distribution of fractures and vugs in fractured-vuggy carbonate reservoirs is highly discontinuous, spanning a wide range of scales. Both free flows and porous flows coexist in these reservoirs, and the internal chemical deposition, entrained fillings, and collapse fillings add to their complexity. Conventional injection and production methods are not fully suitable for such reservoirs. Displacement efficiency evaluation primarily relies on experimental methods, where scaled geological models based on similarity principles are created using AM to aid in the formulation or optimization of displacement strategies. Recently, Wei Y. and Dongxiao Z. [23] developed a visual AM model of fractured-vuggy carbonate reservoirs based on CT scans [Figure 9e], studying the distribution of residual oil and the mechanisms of enhanced oil recovery (EOR) via reverse water injection after water flooding. Jing W. et al. [175] further explored the typical formation mechanisms of residual oil after water flooding [Figure 9f]. Yu-Chen W. et al. [176] compared the displacement effects of foam flooding, water flooding, and gas flooding in fractured-vuggy carbonate reservoirs, focusing on the mechanisms of foam flooding and the distribution characteristics of residual oil [Figure 9f]. These findings underscore the important role of AM in developing displacement strategies and EOR for fractured-vuggy carbonate reservoirs, with broader implications for the study of other complex reservoirs. However, due to limitations in model data, equipment precision, and material properties, AM models are not yet capable of accurately capturing core parameters, such as the mechanical properties, wettability, and surface quality of natural reservoirs and fillings. The complexity and fidelity of these models need further improvement.

## 3. Challenges and Prospects of AM in Oil and Gas Extraction and Gathering Engineering

### 3.1. Overview

AM technology holds great promise for applications in oil and gas extraction and gathering engineering. Overall, the research and application of AM technology in oil and gas extraction and gathering engineering are still in their infancy, facing challenges in areas such as standards, supply, materials, and processes.

In metal AM, DED and PBF are the two most commonly used technologies, which complement each other through differentiation. DED technology, with its high deposition rate and on-site repair capability, is mainly applied to the manufacturing and repair of medium- and large-sized components, such as onshore oil and gas gathering pipelines, pressure vessels, and underwater welding. PBF technology, with its advantages in high precision and complex structure formation, is currently used for producing turbine blades, precision components for downhole tools, and mechanical performance test specimens. Metal AM has already demonstrated its value in the rapid replacement of failed components, equipment repair, and the optimized design of downhole tools. In the future, by integrating advanced technologies, such as robotic control, high-precision online monitoring, and special-environment electrical signal transmission, it is expected to further push the performance limits of petroleum equipment, enabling downhole structural topology optimization and in situ repair in deep-sea and high-sulfur environments.

In non-metal AM, VP and BJ are widely used in the production of reservoir physical models. They have successfully characterized the mechanical properties of natural rock cores, macro-scale pores, and fracture networks in fractured-vuggy carbonate reservoirs. However, due to limitations in their material performance, scanning resolution, and process precision, they are currently unable to accurately characterize micro- and nanoscale pore-throat structures and multi-physics coupling effects. Future efforts should focus on synergistic breakthroughs in MNAM, high-fidelity digital core modeling, and multi-scale material design to achieve full-scale geological property mapping from micro- and nanoscale pore throats to macro-scale fractures, thereby providing a highly reliable physical simulation foundation for oil and gas development.

### 3.2. Certification and Standards

Currently, organizations such as Lloyd’s Register, the American Petroleum Institute, ASTM, and the International Organization for Standardization (ISO) have issued certifications and standards for AM in oil and gas extraction and gathering engineering. These standards aim to address key issues, such as material selection, manufacturing process control, and quality inspection and testing [8,9,10]. However, they lack the traceability of materials and processes, as well as historical data and experience, which are crucial for ensuring the long-term reliability of AM components used in high-temperature, high-pressure, and corrosive environments. The application of AM in this field is still in its early stages, with limited historical data and experience available, making risk assessment and reliability prediction challenging. According to the latest “Global Oil and Gas Additive Manufacturing Market Trends”, AM applications are expected to experience significant growth from 2024 to 2031, with a projected compound annual growth rate (CAGR) of 11.93%, reaching an estimated market value of USD 31 billion by 2030 [25]. Given the promising market outlook, it is crucial to further refine certification guidelines and standards, as well as establish detailed traceability standards for materials and processes and define risk assessment criteria, to ensure the reproducibility, reliability, and quality of AM components in oil and gas extraction and gathering engineering.

### 3.3. AM Solutions for JIT Manufacturing of Failed Parts

The oil and gas extraction and gathering engineering sector involves numerous components and equipment. Companies, such as ConocoPhillips, Baker Hughes, Chevron, and Lincoln Electric, have actively explored the use of JIT manufacturing for failed parts. However, current practices mainly focus on horizontal comparisons between AM and traditional manufacturing methods in terms of the production time and costs, while lacking vertical exploration in areas such as raw material management, production processes, and finished product distribution, which are crucial for JIT manufacturing. In the future, integrating artificial intelligence and machine learning into efficient AM support systems based on extensive production data could enhance production and supply capabilities. On the one hand, AM support software can provide real-time information on raw materials, production progress, and inventory levels. On the other hand, it can enable the centralized management of all production and testing data, ensuring data consistency and traceability, while also offering optimal parameters during the production process to reduce trial-and-error costs and the production time.

### 3.4. Material Development and Process Optimization in AM

With the development of tight, ultra-deep, offshore, and polar oil and gas resources, traditional materials are increasingly inadequate to meet demands, necessitating the development of new materials suitable for extreme environments [179]. Examples include high-strength, corrosion-resistant alloys for deep-sea applications; high-hardness, high-temperature-resistant alloys for ultra-deep wells; and low-viscosity, low-thermal-expansion resins capable of accurately representing the pore-throat structures and connectivity characteristics of artificial cores. The composition of composite powder materials for oil and gas extraction and gathering engineering should comprehensively consider the service environment and the target performance. Based on alloy design principles, special elements are added to enhance mechanical properties in a targeted manner. The optimal composition is determined through a phase diagram analysis, thermodynamic calculations, and process trials. During the dynamic process of melt pool formation, displacement, and solidification, element diffusion, metallurgical fusion, and microstructural transformations occur within the liquid phase, resulting in a uniformly bonded interface. Complex convective phenomena within the melt pool promote the uniform distribution of components, suppress segregation, and improve the interface bonding quality. Process parameters directly influence the physical and chemical properties of the material: a high-heat input facilitates metallurgical fusion but may cause grain coarsening, the evaporation of low-boiling-point elements, and increased residual stress; a low-heat input may lead to poor interlayer metallurgical bonding and component segregation. Therefore, future efforts should focus on process simulation, process monitoring, and data-driven parameter optimization to achieve an integrated design of process, composition, structure, and performance, enhancing the mechanical properties of the alloy deposition layer while avoiding the formation of brittle phases or undesirable microstructures. Additionally, based on extensive historical data and machine learning algorithms, an auxiliary system for material design and process optimization can be developed, integrating virtual testing, performance prediction, and composition optimization to improve the efficiency of new material development and process parameter matching.

### 3.5. AM Technologies for the Repair of Oilfield Equipment

Currently, the integration of reverse engineering and AM technologies has made pipeline and drill bit repair a reality, with potential applications for other oil equipment repairs as well. These technologies offer efficient and customized repair solutions for both onshore and offshore oil facilities. However, the high reliance on manual labor remains a challenge, and improvements in the repair quality and consistency are needed. Additionally, in situ wellbore rehabilitation presents significant challenges due to the harsh service environment (180 °C, 40 MPa, 150 mm) and limited operational space. Issues such as integrating multi-technology systems, high-precision positioning and scanning, maintaining operational environments, and difficulties in signal transmission complicate the process. In the future, it will be essential to reduce the dependence on manual intervention in critical stages, like 3D scanning, damaged model processing, and setting process parameters, to ensure the quality and consistency of the repaired components. For repairs of oil equipment in extreme environments, the development of core technologies is necessary, including high-precision scanning, intelligent planning of repair paths, and remote manual/automatic repair and remanufacturing. In terms of in situ wellbore rehabilitation, integrating robotic, AM, and petroleum engineering techniques could enable the development of in situ additive repair equipment to precisely address damage to oil pipes, casings, and downhole tools.

### 3.6. Optimization of Downhole Tools Using AM

Downhole tools and equipment are critical for oil production, playing key roles in drilling, logging, completion, and maintenance. Due to the complex wellbore environment, most are hydraulically driven, with control functions typically achieved through components with specialized internal flow paths. However, the high manufacturing costs of these flow path components often require performance trade-offs to reduce costs [21,180]. In contrast to traditional manufacturing methods, AM offers flexible shaping processes, presenting clear advantages in producing specialized components. In the future, optimizing downhole tools through topology optimization and AM is expected to become a trend. The structure–function relationship of downhole tools, such as drill bits, core retrieval tools, workover tools, and packers, will become increasingly integrated. Additionally, the integration of components, similar to practices in the aerospace sector, can be adapted to improve downhole tools. As multi-material AM advances, it will enable the deposition of wear-resistant, high-hardness, and impact-resistant materials at key locations, such as perforating tool nozzles, shock hammer components, and the interior cavities of hydrocyclones, thereby further extending the tools’ life and expanding their application range.

## 4. Conclusions

AM technologies, such as PBF, DED, VP, BJ, and MNAM, have been explored and applied in oil and gas extraction and gathering engineering, achieving early successes and demonstrating significant potential with broad prospects. Among these, PBF and DED technologies are widely used in the manufacturing and repair of oil and gas pipelines, related components, and downhole tools and equipment. DED is primarily utilized for the production and repair of large components due to its versatility, while PBF is favored for the precise detailing of smaller components because of its high precision and density. VP, BJ, and MNAM are typically applied in mold production, as well as in the fabrication of artificial cores and reservoir geological models.

From a just-in-time (JIT) manufacturing perspective, AM plays a crucial role in ensuring continuous production in extreme or offshore environments, as critical spare parts no longer need to occupy significant storage space. Instead, maintaining a supply of raw materials is sufficient, significantly reducing storage pressures.

However, the current application of AM in oil and gas extraction and gathering is still in its early stages, facing challenges such as the certification of AM components, process and product standardization, and limited material options. Future development trends include the following: (1) improving certification and standards, refining material and process traceability, and enhancing risk assessment guidelines; (2) developing AM support systems to boost JIT manufacturing and supply capabilities; (3) utilizing AM for petroleum equipment repair to extend the service life and improve performance; (4) developing new materials tailored to various application scenarios in oil and gas extraction and gathering; and (5) optimizing petroleum equipment design by fully leveraging the flexibility of AM.

In summary, as an emerging manufacturing technology, AM offers several advantages, including high flexibility, precision, rapid prototyping, and the ability to repair specialized components. These capabilities make it particularly suitable for oil and gas extraction and gathering engineering, enabling the JIT manufacturing of failed parts, reducing inventory pressures for critical spare parts, shortening manufacturing and repair cycles, and extending the lifespan and performance of equipment. While challenges remain in terms of standards, materials, processes, and costs, ongoing technological advancements will undoubtedly expand AM’s role in oil and gas extraction and gathering, presenting both new opportunities and challenges for the petroleum sector.

## Figures and Tables

**Table 1:** Summary of materials processed using different AM technologies and their application scenarios [27,28,29,30,31,32].

Type			Formable Materials	Existing/Potential Applications
Binder jetting (BJ)			Metal, ceramic, sandstone, polymer, composites	1. Impeller mold. 2. Rapid prototyping.
Directed energy deposition (DED)	Laser wire welding additive manufacturing (LWWAM)		Metal powder/wire	1. Drill bit repair. 2. Pump casing and valve repair.
	Laser-assisted directed energy deposition (LDED)			1. Functional coatings. 2. Impeller repair.
	Wire arc additive manufacturing (WAAM)			High-pressure pipeline assembly.
Material extrusion (ME)	Fused deposition modeling (FDM)		Thermoplastic, metal, composites	Rapid prototyping.
Material jetting (MJ)	Material jetting (MJ)		Thermoplastic	1. Reservoir geological model. 2. Rapid prototyping.
	Nano particle jetting (NPJ)		Metal	
	Drop-on-demand (DOD)		Wax	
Powder bed fusion (PBF)	Laser powder bed fusion (LPBF)	Selective laser melting (SLM)	Metal	1. Impeller direct forming. 2. Pump casing and valve.
		Selective laser sintering (SLS)	Polymers, metal, composites	
	Electron beam melting (EBM)		Metal	
Sheet lamination (SL)	Laminated object manufacturing (LOM)		Paper, plastic film, metal	1. Large equipment prototypes. 2. Sensor protection covers.
	Ultrasonic additive manufacturing (UAM)		Metal	
Vat polymerization (VP)	Stereolithography (SLA)		Photopolymer resin, ceramics	1. Rapid prototyping. 2. Artificial core. 3. Reservoir geological model.
	Digital light processing (DLP)			
	Continuous digital light processing (CDLP)			
	Continuous liquid interface production (CLIP)			

**Table 2:** Literature review on multi-metal PBF and its potential applications in oilfields.

Issues of Concern	Material	Existing Issues	Solutions	Potential Applications
Material compatibility	AlSi10Mg/AlCuNiFeMg (SLM) [42]	Parameters cannot match the thermal properties of different materials.	1. Selection of compatible materials. 2. Optimization of printing parameters. 3. Improvement of post-processing.	High-strength, corrosion-resistant manifold for heat exchangers.
	316L/Cu10Sn (SLM) [43]	Steel and copper’s differing thermal conductivities cause interface protrusion.		
Pore and crack	316L/Cu10Sn (SLM) [44]	The gas in the molten pool is not fully released.	1. Optimization of printing parameters. 2. Use of support structures. 3. Improvement of post-processing.	1. High-strength, wear-resistant drill tools. 2. Piston pump cylinder bodies and pistons.
	Fe/Al (SLM) [45]	Interlayer thermal gradients cause localized deformation or cracking.		
	SS316/SiC/TiN (SLS) [46]	Cracks result from high temperature gradients, poor wettability, and high-energy-density sintering.		
Alloy element segregation/loss	Ni/Ti (SLM & EBM) [47]	Particle–particle and particle–gas interactions cause significant Ni evaporation, leading to material embrittlement.	1. Optimization of printing parameters. 2. Use of pre-mixed powder. 3. Improvement of post-processing.	1. Downhole safety valve. 2. Corrosion-resistant valve.
Oxide/compound inclusions	316L/C52400 (SLM) [48]	Oxides in the melt pool caused cracking on the 316L side.	1. Creating inert/reducing atmosphere. 2. Incorporation of special materials. 3. Improvement of post-processing.	Corrosion-resistant, high-pressure vessel.
	Cu10Sn/Ti6Al4V (SLM) [49]	High hardness intermetallic (TiCu and Cu1000Ti) cause interlayer cracking.		
Interface phases and unmelted particles	Al12Si/Al3.5Cu1.5Mg1Si (SLM) [50]	The eutectic Al-Si structure and discontinuous microstructure result in lower hardness.	1. Interfacial transition is layer-assisted. 2. Multi-laser control. 3. Improvement of post-processing.	1. Impact-resistant drill bit. 2. Corrosion-resistant pipe joint.
Corrosion/wear resistance	SS316/SiC/TiN (SLS) [46]	Unreasonable parameters can increase porosity and wear rate.	1. Chemical passivation. 2. Plasma nitriding. 3. Electroless plating.	Corrosion and wear-resistant coatings for drill pipes and drill tools.
	AISI 430 Steel/Inconel 625 (EBM) [51]	Rapid solidification can cause cracks and increase localized corrosion risk.		

**Table 3:** Cost comparison between typical AM parts and traditionally manufactured parts [71].

Geometry	Dimensions (mm)	Mass (kg)	Traditional Cost (USD)	AM Cost (USD)	Cost Increase (%)
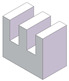	40 × 45 × 27	2.4	618	923	50
	60 × 68 × 38.5	3.2	966	1231	27
	80 × 90 × 54	6	1523	2309	52
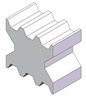	40 × 45 × 27	2.4	786	923	17
	60 × 68 × 38.5	3.2	1140	1231	8
	80 × 90 × 54	6	2188	2309	5
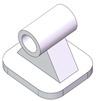	45 × 50 × 21.5	1.6	1416	617	−56
	67.5 × 75 × 27.25	2.4	2184	923	−58
	90 × 100 × 43	6	2762	2309	−16

**Table 4:** Corrosion resistance of AM steel in marine environments.

Material	Main Conclusions on Corrosion Resistance
304L and 316L (PBF) [87]	Less prone to atmospheric pitting and intergranular corrosion than forged parts.
316L (not stated) [88]	Improved microstructure with enhanced fretting corrosion resistance.
316L (SLM) [89]	Increased pitting potential, lower passivation and corrosion current densities.
K-81TW (WAAM) [90]	Improved microstructure and texture, with better corrosion resistance.
309L (WAAM) [91]	Higher pitting potential, coating thickness positively correlates with corrosion resistance.
A36 (WAAM) [92]	Slightly higher corrosion rate, but still lower than forged steel.

**Table 5:** Mechanical optimization through residual stress and micro-defect elimination.

Improvement Method	Materials	Enhancement Effects	Potential Applications
Recrystallization stress relief	Ti6Al4V (DED)	Kernel average misorientation (KAM) reduction [115].	Compressor rotor
	24CrNiMo (PBF)	Residual stress: tensile to compressive [116].	High-pressure oil and gas separator
	Inconel 718 (PBF)	Yield strength increased by 8.72% [117].	Acid gas treatment
	Ni-15Fe-5Mo (PBF)	Magnetic properties exhibit anisotropy [118].	Precision sensor housing
Microstructure control	FeCrMoBC (DED)	Hardness adjustable from 650 to 1400 HV [119].	Large bearings/gears
	2209 DSS (DED)	Adjustable hardness (301–327 HV), impact toughness (118–154 J), tensile strength (750–790 MPa) [120].	Offshore platform structural components
	316L/Inconel 625 (DED)	Balanced strength (675.64 MPa) and ductility (33.6%) [121].	Large chemical containers
	Q235 (DED)	Ultra-high strength (961 ± 40 MPa), good ductility (37.5 ± 3%), thermal stability at 500 °C [122].	Chemical reactor
	316L (DED)	Reduced tensile strength anisotropy, improved ductility [123].	Heat exchanger
	18Ni-300 (PBF)	Increase in ductility by 30–35%, fracture toughness improvement by over 50% [124].	1. High-pressure gas cylinders. 2. Hydrogen storage tanks.
	Ti6Al4V (PBF)	Simultaneous improvement of strength and ductility [125].	1. High-pressure pipeline components. 2. Deep-sea oil and gas extraction equipment. 3. High-pressure reactors.
		Cyclic fatigue strength: 714 MPa [126].	
		Fatigue life doubled under high strain [127].	
Temperature gradient control	IN718 (DED)	High hardness (500 HV), high tensile strength (1284 MPa) [128].	High-temperature/pressure valve
	ERCuAl-A2 ER-120S-G (DED)	Tensile strength: 690 MPa, elongation at break: 16.6% [129].	High-pressure boiler
	W6Mo5Cr4V2 (PBF)	High hardness (60 HRC) and high tensile strength (1000 MPa) [130].	Offshore wellhead equipment
	Ni10Cr6W1Fe9Ti1 (PBF)	Tensile strength: 961.65 MPa; yield strength: 739.77 MPa; elongation: 26.5% [131].	Deep-sea drilling equipment

**Table 6:** Patents involving AM components for downhole tools.

Company	Application	AM-Produced Parts
Halliburton	Plugging tools	Plastic, ceramic, and metal sealing components [151]
	Drilling equipment	Hard coatings on drill bits [152]
	Custom tooling	Antennas, circuit boards, caliper arms in logging tools [153]
Baker Hughes	Detectors	Detector hatch and housing [154]
		Insulated support frame [155]
	Downhole tools	Parts with internal special flow channel structures [156]
		Directly formed sealing elements on sliding sleeves [157]
	Electrical submersible pump	Bearing housings and bushings [158]
	Metal corrosion prevention	Neutralizing media and medium containers for corrosion protection [159]
	Drilling equipment	Wear-resistant inserts for drill bits [160]
Schlumberger	Downhole tools	Circuit and multilayer electrical system integration [161]
Spartan	Detectors	Multilayer labyrinth housings [162]

**Table 7:** Overview of MNAM process [24].

Process Classification		MNAM Technique	Minimum Feature Size	Advantages	Disadvantages
VP	Single-photon	μSLA	≥1.2 μm	1. Large parts can be built easily. 2. High accuracy.	1. High cost and high equipment requirements. 2. Low printing efficiency.
		PμSL	≥0.6 μm	1. Faster than μSLA. 2. High accuracy.	Molding size is smaller than the μSLA due to the limitation of DMD resolution.
		CLIP	≥7.6 μm	Faster than μSLA and PμSL.	Selected release film has high requirements for both oxygen and light transmission.
		Micro-CAL	≥20 μm	3D projection in comparison with μSLA, PμSL, and CLIP.	Low accuracy in comparison with μSLA, PμSL, and CLIP.
	Two-photon	TPP	≥18 μm	Highest resolution MNAM process.	Costly equipment due to the need for highly accurate optics and positioning stage.
ME	DIW	——	≥1 μm	Compatible with a wide range of materials.	Minimum feature size limited by nozzle diameter and material properties.
	FDM	——	≥45 μm	Inexpensive printers and filaments can be integrated to achieve multi-material printing.	1. Supporting structures required for free-standing models. 2. Limited printing accuracy.
MJ	EHD jet printing	——	≥80 μm	Nanoscale fibers can be fabricated.	Precise control over deposition difficult with far-field EHD.
	AJ deposition	——	≥5 μm	Printing in multiple directions with a wide range of materials.	Printing efficiency is limited by ink solids content and particle size.
Other	Micro-SLS	——	≥5 μm	1. Fabricate high precision metal 3D microstructures. 2. No need for supporting structures or supporting materials.	1. Higher demands on optical systems, displacement stages, powder particle size. 2. High surface-finishing requirements.
	Micro-SLM	——	≥15 μm	——	——
	ECAM	——	≥0.3 μm	High accuracy and high surface finishes.	Slow build process and poor mechanical properties.

## Data Availability

No new data were created or analyzed in this study. Data sharing is not applicable to this article.

## Abbreviations

The following abbreviations are used in this manuscript:

AM	additive manufacturing
JIT	just-in-time
BJ	binder jetting
DED	directed energy deposition
LWWAM	laser wire welding additive manufacturing
LDED	laser-assisted directed energy deposition
WAAM	wire arc additive manufacturing
ME	material extrusion
FDM	fused deposition modeling
MJ	material jetting
NPJ	nano particle jetting
DOD	drop-on-demand
PBF	powder bed fusion
LPBF	laser powder bed fusion
SLM	selective laser melting
SLS	selective laser sintering
EBM	electron beam melting
SL	sheet lamination
LOM	laminated object manufacturing
UAM	ultrasonic additive manufacturing
VP	vat polymerization
SLA	stereolithography
DLP	digital light processing
CDLP	continuous digital light processing
CLIP	continuous liquid interface production
FGM	functionally graded material
API	American Petroleum Institute
ASTM	American Society for Testing Materials
ISO	International Organization for Standardization
ESP	electrical submersible pump
FPSO	floating production storage and offloading
NAB	nickel aluminum bronze
UDMD	underwater direct metal deposition
ROV	remotely operated vehicle
KAM	Kernel average misorientation
DNV	Det Norske Veritas
FFD	full factorial design
CFD	computational fluid dynamics
EOR	enhanced oil recovery
MNAM	micro/nano additive manufacturing
MEMS	microelectromechanical systems
PµSL	projection microstereolithography
CAGR	compound annual growth rate
